# The Serum MicroRNA Signatures for Pancreatic Cancer Detection and Operability Evaluation

**DOI:** 10.3389/fbioe.2020.00379

**Published:** 2020-04-29

**Authors:** Qiuliang Yan, Dandan Hu, Maolan Li, Yan Chen, Xiangsong Wu, Qinghuang Ye, Zhijiang Wang, Lingzhe He, Jinhui Zhu

**Affiliations:** ^1^Department of General Surgery, Jinhua People’s Hospital, Jinhua, China; ^2^Department of General Surgery, Second Affiliated Hospital, Zhejiang University School of Medicine, Hangzhou, China; ^3^Department of General Surgery, Xinhua Hospital, Shanghai Jiao Tong University School of Medicine, Shanghai, China

**Keywords:** pancreatic cancer, diagnosis, liquid biopsy, miRNAs, functional analysis

## Abstract

Pancreatic cancer (PC) has high morbidity and mortality. It is the fourth leading cause of cancer death. Its diagnosis and treatment are difficult. Liquid biopsy makes early diagnosis of pancreatic cancer possible. We analyzed the expression profiles of 2,555 serum miRNAs in 100 pancreatic cancer patients and 150 healthy controls. With advanced feature selection methods, we identified 13 pancreatic cancer signature miRNAs that can classify the pancreatic cancer patients and healthy controls. For pancreatic cancer treatment, operation is still the first choice. But many pancreatic cancer patients are already inoperable. Therefore, we compared the 79 inoperable and 21 operable patients and identified 432 miRNAs that can predict whether a pancreatic cancer patient was operable. The functional analysis of the 13 pancreatic cancer signatures and the 432 operability miRNAs revealed the molecular mechanisms of pancreatic cancer and shield light on the diagnosis and therapy of pancreatic cancer in clinical practice.

## Introduction

Pancreatic cancer (PC) has high morbidity and mortality. It is the fourth leading cause of cancer death (Siegel et al., 2013). It also has strong invasiveness and metastasis, and has a high tolerance to chemotherapy, resulting in a low survival rate (Vogel and Tommaso, 1990; Cheng et al., 2012; Tamburrino et al., 2013). At present, surgical resection is still the most effective treatment strategy. It is worth noting that <25% of the potentially localized tumors can be removed, and surgical removal seems to be more suitable for patients with early PC (Li et al., 2004; Seppänen et al., 2017). For locally advanced, non-resectable and metastatic PC, treatment is palliative. Therefore, the research on the early diagnosis of PC is particularly important.

PC is hidden behind other abdominal organs, and the lack of detectable special symptoms makes early diagnosis difficult (Hingorani et al., 2003). In addition, the lack of effective diagnostic tools also led to a low 5-year survival rate, from more than 50% in stage I to <5% in advanced patients (Rosty and Goggins, 2002; Wagner et al., 2004). In general, routine diagnostic methods include imaging and serum markers (Frič et al., 2016). However, conventional imaging methods such as CT and MRI tend to work only after the emergence of local and systemic symptoms occurred (Costello et al., 2012). Serum markers such as carbohydrate antigen 19-9 (CA19-9) and carcinoembryonic antigen (CEA) can’t detect precancerous and early lesions because of their low sensitivity and specificity, so they are often used to monitor postoperative reactions and judge prognosis (DiMagno et al., 1999; Chang and Kundranda, 2017). To this end, we are committed to develop highly sensitive and specific non-invasive biomarkers to improve the diagnosis of PC.

In recent years, many studies have shown that blood-based miRNAs can be used as potential biomarkers for tumor diagnosis (Chen et al., 2008; Ebrahimi et al., 2016; Huang et al., 2016). These serum miRNAs expression patterns have the potential to identify a variety of human cancers, including breast cancer (McAnena et al., 2019), ovarian cancer (Zhu et al., 2017), and gastric cancer (Li Y. et al., 2019). There are also studies exploring the significance of serum miRNA in the diagnosis of PC (Madhavan et al., 2015; Subramani et al., 2015). These studies suggest that miRNAs may be used as serum markers for early diagnosis of PC. In this study, we analyzed the serum miRNA expression profiles of patients with PC and normal subjects in order to screen the miRNA that can be used to diagnose pancreatic cancer. At the same time, we also showed that some miRNA can evaluate the surgical feasibility of patients with PC.

## Methods

### The Serum miRNA Expression Profiles of Pancreatic Cancer Patients

The expression profiles of 2,555 serum miRNAs in 100 pancreatic cancer patients and 150 healthy controls were downloaded from GEO (Gene Expression Omnibus) database under accession number of GSE59856 (Kojima et al., 2015). Within the 100 pancreatic cancer patients, there were 79 inoperable and 21 operable patients. The clinical information of these patients was given in Supplementary Table S1. There were 27 cStage III, 54 cStage IV, 3 pStage IIA, 14 pStage IIB, 1 ypStage IB, 1 ypStage IIB patients. The age ranged from 33 to 82 with median of 67. There were 36 female and 64 male patients. In the original dataset, there were some missing values. For each miRNA, the largest missing value percentage was 0.5%. We imputed these missing values using k-Nearest Neighbor (KNN, *K* = 3) method in R/Bioconductor package impute^1^ (Troyanskaya et al., 2001). Our goal was not only to find the pancreatic cancer miRNAs that were different between pancreatic cancer patients and healthy controls, but also to identify the operability miRNAs that were different between inoperable and operable patients.

### The Pancreatic Cancer miRNA Signature Identification

There have been many differential expression analysis methods. For example, *t*-test *p*-value and fold change are most widely used to identify differentially expressed genes. But such methods do not consider the relationship between features, i.e., miRNAs in this study. Therefore, these methods will get a lot of redundant features. Many genes with the same trend will be selected and when the number of genes is too large, they are not suitable as signatures. In this study, we applied the state-of-art feature selection methods to identify the pancreatic cancer miRNA signatures.

First, the Monte Carlo feature selection (MCFS) (Jiang et al., 2019; Li J. et al., 2019; Pan et al., 2019b; Chen et al., 2020) was used to rank the miRNAs based on their relative importance (RI) (Draminski et al., 2008) which was calculated by considering how much this miRNA participated in classifying the samples in a serial of decision trees constructed on subsets of the data. All the 2,555 serum miRNAs can be ranked. The top ranked miRNAs had strong classification power. Therefore, with few top ranked miRNAs, we should be able to classify the samples very well. The dmLab software downloaded from http://www.ipipan.eu/staff/m.draminski/mcfs.html with default parameters (Draminski et al., 2008) were used to apply the MCFS algorithm.

Then, we used incremental feature selection (IFS) (Zhang et al., 2016; Zhou et al., 2016; Li and Huang, 2018; Chen et al., 2019a; Li J. et al., 2019; Pan et al., 2019a) to identify the pancreatic cancer miRNA signature. The top 1, top 2, top 3, … top 600 miRNAs were selected and tested. Each time, support vector machine (SVM) classifiers were built and the accuracy was estimated with leave-one-out cross validation (LOOCV) (Huang et al., 2012, 2013). The number of miRNAs with the best performance, i.e., the highest LOOCV accuracy, was identified as pancreatic cancer miRNA signature.

### The Operability miRNA Signature Identification

Similarly, we can use MCFS and IFS feature selection methods to identify the operability miRNA signatures by changing the samples of 100 pancreatic cancer patients and 150 healthy controls with 79 inoperable and 21 operable patients. Another difference was that the sample sizes of 79 inoperable and 21 operable patients were seriously imbalanced. Therefore, we used Matthew’s correlation coefficient (MCC) (Chen et al., 2017, 2019b; Pan et al., 2018) instead of accuracy to evaluate the prediction performance. MCC was defined as

(1) $$M\,C\,C=\frac{T\,P\times T\,N-F\,P\times F\,N}{\sqrt{\left(T\,P+F\,P\right)\,\left(T\,P+F\,N\right)\,\left(T\,N+F\,P\right)\,\left(T\,N+F\,N\right)}}$$

in which TP, TN, FP, and FN were the numbers of true positive, true negative, false positive and false negative samples, respectively.

When the sizes of negative and positive samples are imbalanced, if we predict all samples to be the dominate class, the accuracy is high but the model is usefulness. To overcome this problem, we need to balance the sensitivity and specificity and MCC becomes a better metric than accuracy (Chen et al., 2017; Pan et al., 2018; Chen et al., 2019b). As shown in Equation (1), MCC considered not only how well positive samples were predicted but also how well the negative samples were predicted. TP, TN, FP, and FN were all taken into consideration.

## Results

### The Identified Pancreatic Cancer miRNA Signatures

**FIGURE 1:**
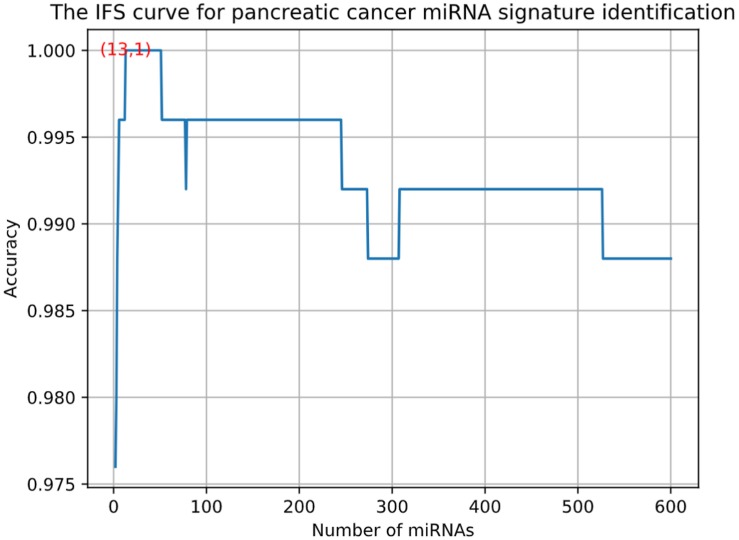
The IFS curve for pancreatic cancer miRNA signature identification. The x-axis was the number of miRNAs while the y-axis was LOOCV accuracy. It can be seen that with 13 miRNAs, all the samples can be perfectly predicted.

**FIGURE 2:**
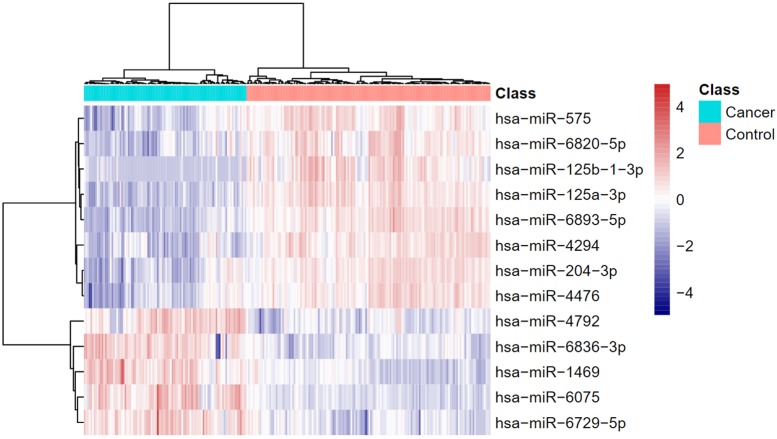
The heatmap of pancreatic cancer miRNA signature. The 13 pancreatic cancer miRNA signatures can perfectly classify the pancreatic cancer and healthy controls even with simple hierarchical clustering.

**FIGURE 3:**
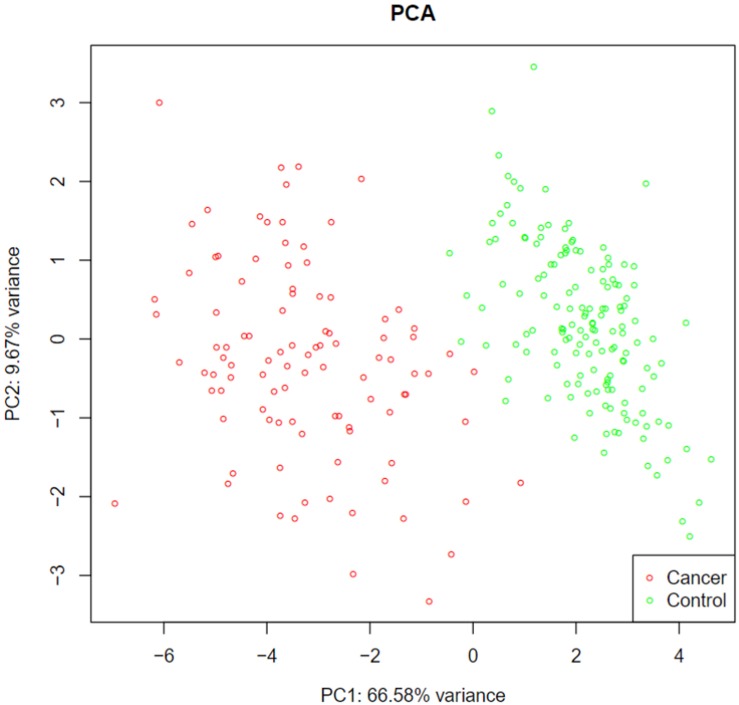
The PCA plot of pancreatic cancer miRNA signature. The first principal component (PC1) which represented 66.58% variance of the pancreatic cancer miRNA signature can clearly group the cancer samples and healthy controls into two parts.

Using advance feature selection methods of MCFS and IFS, we identified the pancreatic cancer miRNA signatures and meanwhile constructed the pancreatic cancer predictor based on these miRNAs. The IFS curve with the number of miRNAs as x-axis and the LOOCV accuracy as y-axis was shown in Figure 1. It can be seen that with 13 miRNAs, all the samples can be perfectly predicted. Of course, we should be aware that this accuracy was the results of wrapped feature selection analysis and was primarily used to evaluate how much the selected features differed between sample groups. It should not be considered as the final prediction model which needed to be validated on independent large cohort data. The 13 pancreatic cancer signature miRNAs were listed in Table 1. We plotted the heatmap of the 100 pancreatic cancer patients and 150 healthy controls using the 13 miRNAs (Figure 2). It can be seen that the 13 pancreatic cancer miRNA signatures can perfectly classify the pancreatic cancer and healthy controls even with simple hierarchical clustering. We also performed Principal Component Analysis (PCA) and plotted the PCA plot with the first principal component (PC1) and the second principal component (PC2) in Figure 3 and PC1 which represented 66.58% variance of the pancreatic cancer miRNA signature can clearly group the cancer samples and healthy controls into two parts.

**TABLE 1:** The 13 pancreatic cancer signature miRNAs.

Rank	miRNA	RI
1	hsa-miR-125a-3p	0.757
2	hsa-miR-6893-5p	0.647
3	hsa-miR-125b-1-3p	0.572
4	hsa-miR-6075	0.524
5	hsa-miR-6836-3p	0.512
6	hsa-miR-1469	0.460
7	hsa-miR-6729-5p	0.449
8	hsa-miR-575	0.437
9	hsa-miR-204-3p	0.409
10	hsa-miR-6820-5p	0.350
11	hsa-miR-4294	0.347
12	hsa-miR-4476	0.317
13	hsa-miR-4792	0.303

To further explore the functions of the identified miRNAs, we searched their targets in miRDB^2^ (Wong and Wang, 2015). The results were listed in Supplementary Table S2. Except hsa-miR-4792 which was not included in miRDB, all the other 12 miRNAs’ targets were predicted. Since there were usually many predicted targets, the target genes were ranked based on target score. The higher target score was, the more likely it was an actual target. The top ranked genes had high confidence to be true.

**FIGURE 4:**
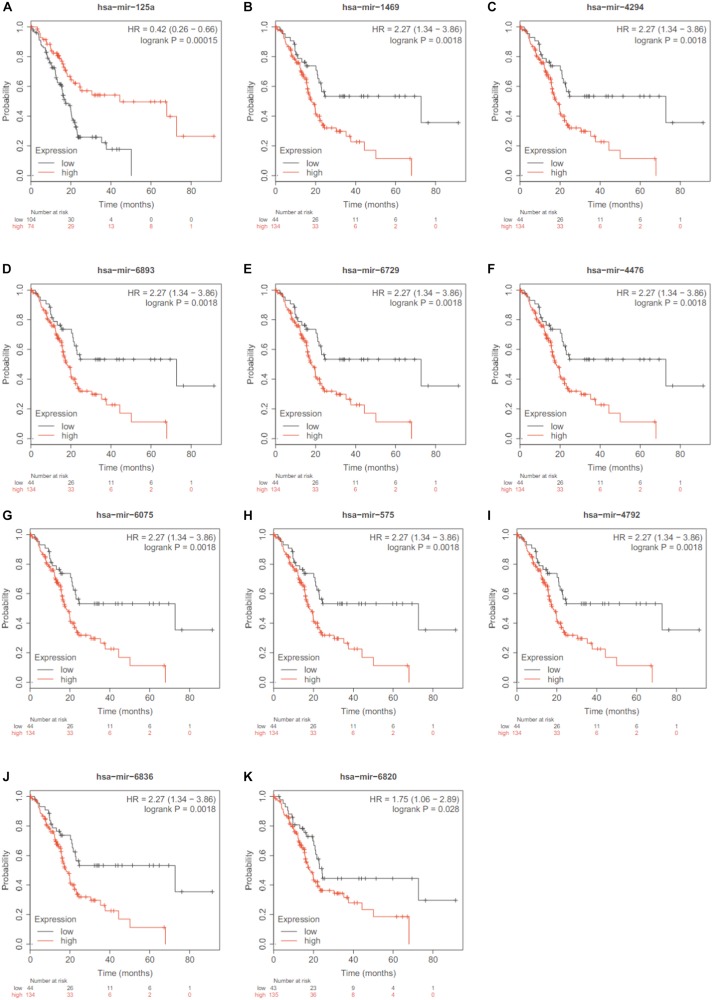
The KM plots of the 11 significant miRNAs. Except hsa-miR-125b and hsa-miR-204, 11 out of the 13 identified miRNAs were significantly associated overall survival of pancreatic ductal adenocarcinoma based on Kaplan Meier-plotter. **(A)** hsa-mir-125a; **(B)** has-mir-1469; **(C)** has-mir-4294; **(D)** V has-mir-6893; **(E)** has-mir-6729; **(F)** has-mir-4476; **(G)** has-mir-6075; **(H)** has-mir-575; **(I)** has-mir-4792; **(J)** has-mir-6836; **(K)** has-mir-6820.

Since the association with survival was a strong evidence of the miRNA importance, we searched the 13 miRNAs in Kaplan Meier-plotter^3^ (Nagy et al., 2018). Except hsa-miR-125b and hsa-miR-204, 11 out of the 13 identified miRNAs were significantly associated overall survival of pancreatic ductal adenocarcinoma. Their Kaplan Meier (KM) plots were shown in Figure 4.

**FIGURE 5:**
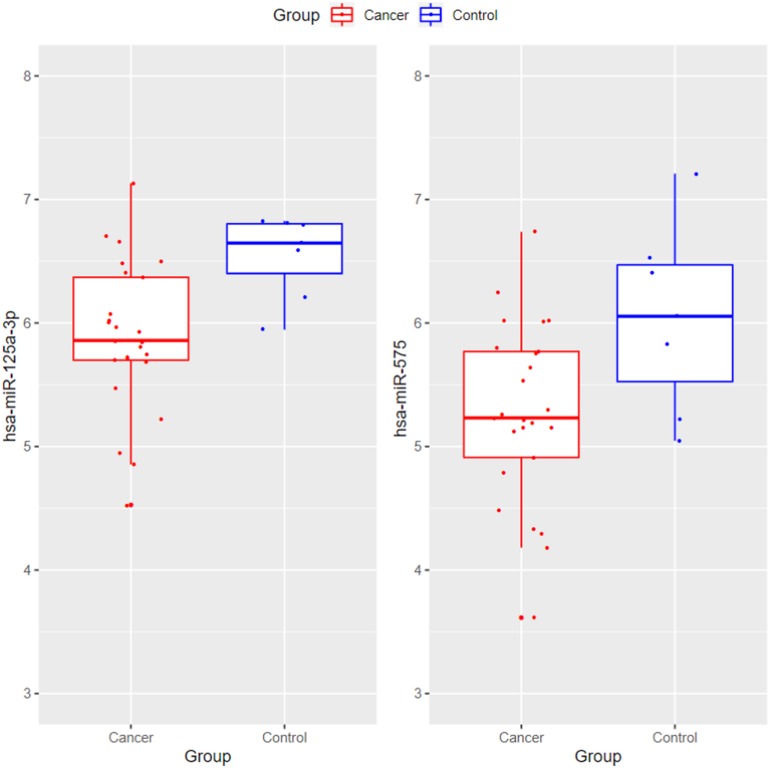
The boxplots of hsa-miR-125a-3p and hsa-miR-575 between pancreatic cancer and control tissues. The *t*-test *p*-values of hsa-miR-125a-3p and hsa-miR-575 between cancer and control tissues were 0.00165 and 0.0384, respectively. Their expression levels were significantly lower in cancer tissues than in control tissues. The tissue results were consistent with our serum results.

The blood may have different expression pattern with tissue. Therefore, we wanted to compare the blood and tissue expression profiles of the 13 identified miRNAs. The GSE32678 miRNA expression profiles (Donahue et al., 2012) which included 25 pancreatic cancer tissue samples and 7 control tissue samples were used for comparison. Since the miRNA platforms were different cross datasets, there were two overlapped miRNAs: hsa-miR-125a-3p and hsa-miR-575. Their boxplots were shown in Figure 5. The *t*-test *p*-values of hsa-miR-125a-3p and hsa-miR-575 between cancer and control tissues were 0.00165 and 0.0384, respectively. Their expression levels were significantly lower in cancer tissues than in control tissues. The tissue results were consistent with our serum results as shown in Figure 2.

### The Identified Operability miRNA Signatures

**FIGURE 6:**
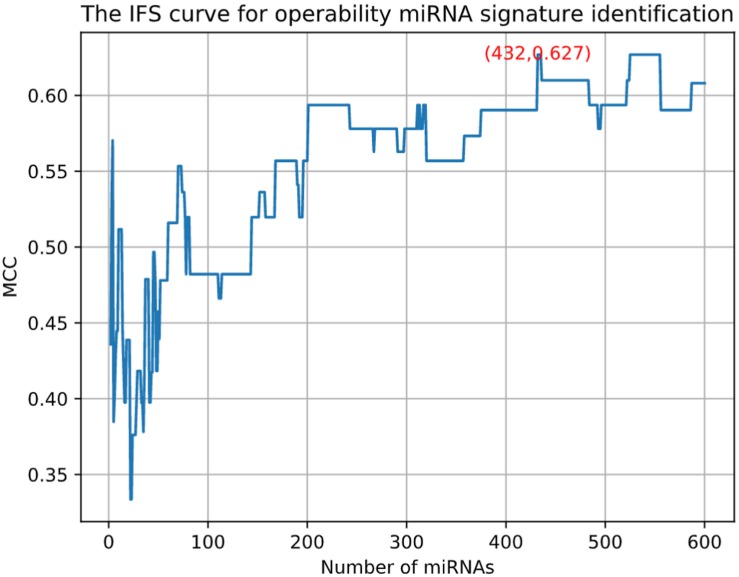
The IFS curve for operability miRNA signature identification. The x-axis was the number of miRNAs. Since the sample sizes of inoperable and operable patients were highly imbalanced, the y-axis was MCC which considered both sensitivity and specificity.

Pancreatic cancer has high mortality. Most of the pancreatic cancers are inoperable. Therefore, we want to identify the miRNAs that can predict whether a pancreatic cancer patient is operable. Similarly, we used MCFS and IFS methods to do the feature selection and identified 432 miRNAs. Unlike the previous IFS curve in Figure 1, the IFS curve of operability miRNA signature identification (Figure 6) used MCC as y-axis since the sample sizes of inoperable and operable patients were highly imbalanced. It can be seen that with 432 miRNAs, the MCC was the highest, 0.627. The 432 operability signature miRNAs were listed in Supplementary Table S3. Meanwhile, we also calculated sensitivity, specificity and accuracy based on the confusion matrix of actual and predicted results as shown in Table 2. The sensitivity, specificity and accuracy were 0.857, 0.848, and 0.850, respectively. The operability prediction was much harder than pancreatic cancer prediction. Even with 432 miRNAs, the prediction performance was still much worse than pancreatic cancer prediction.

**TABLE 2:** The confusion matrix of actual and predicted operable and inoperable patients.

	Predicted operable patients	Predicted inoperable patients
Actual operable patients	18	3
Actual inoperable patients	12	67

## Discussion

We analyzed the serum miRNA expression profiles of 100 PC samples and 150 normal samples by bioinformatics. Fortunately, we found 13 serum miRNA (miR-125a-3p, miR-6893-5p, miR-125b-1-3p, miR-6075, miR-6836-3p, miR-1469, miR-6729-5p, miR-575, miR-204-3p, miR-6820-5p, miR-4294, miR-4476, and miR-4792) that can perfectly distinguish PC patients from normal subjects. By comparing the existed studies of these miRNA in PC, the availability of our screening methods and the significance of the screening results are explored.

MiR-125a-3p is considered to be a down-regulated tumor suppressor factor in PC cells (Moriya et al., 2017). After further study by Liu et al. (2018), it was found that miR-125a-3p could directly inhibit the expression of Fyn, thus promoting the EMT process of PC, and the overexpression of Fyn could partially reverse the drug sensitivity of miR-125a-3p to gemcitabine. Kojima et al. (2015) used gene chip technology to develop a diagnostic index to distinguish PC from other clinical conditions, including eight miRNAs. This study showed that the sensitivity, specificity and accuracy of these miRNA were 80.3, 97.6, and 91.6%, respectively, which were higher than those of CA19-9 (65.6, 92.9, and 82.1%) and CEA (40.0, 88.6, and 71.8%). Five out of the eight miRNAs (miR-125a-3p, miR-6075, miR-6836-3p, miR-4294, and miR-4476) (Kojima et al., 2015) were also identified by us. The study on the application of miRNA in the diagnosis of PC shows that the evaluation of these miRNA markers is of clinical value, and the side also proves the feasibility and clinical value of our findings.

We further divided 100 PC samples into 21 operable and 79 inoperable, and screened out some differential miRNA. We compared whether these miRNAs are included in Pancreatic Cancer Database^4^ (Thomas et al., 2014) and analyze the research value of these miRNAs in PC.

Zou et al. (2019) have also shown that miR-19a-3p is significantly up-regulated in PC and can be used for early and non-invasive diagnosis of PC. In addition, the increase of serum miR-19a-3p was closely related to the poor overall survival (OS) rate (Zou et al., 2019). MiR-30a-3p was also discovered to inhibit cancer by controlling the expression of p27 in PC (Kim et al., 2018), and miR-17-3p was significantly increased in pancreatic cysts and could be used as a potential biomarker of cystic precursor lesions of PC (Ryu et al., 2011). As a potential prognostic marker of PC, miR-4521 was found to be enriched in various biological processes such as cell proliferation and cell cycle, and was associated to OS in PC (Liao et al., 2018). According to Zhou B et al., miR-655 could inhibit the expression of TGF-BR-1 and TGF-BR-2, thus reducing the invasiveness of PC and improving the feasibility of operation (Zhou et al., 2018). It was reported by Yang et al. (2019) that miR-144-3p could target MMP7 then regulate the malignant progression of PC, suggesting that patients with high expression of miR-144-3p have longer survival time. These studies show that many members of the 432 miRNAs we screened have shown their relationship with malignant progression of PC and are closely related to the OS of patients. Biological processes related to malignant progression, such as cell proliferation, invasion and migration, are often closely related to the feasibility of surgical resection in patients with PC. In other words, our study on the expression of miRNA in 21 operable PC samples and 79 inoperable PC samples is meaningful, and the selected miRNAs used to evaluate the surgical feasibility of PC patients also has clinical value.

## Data Availability Statement

All datasets generated for this study are included in the article/Supplementary Material.

## Author Contributions

JZ, QYa, DH, ML, YC, XW, QYe, ZW, and LH contributed to the study design. QYa, DH, XW, and YC conducted the literature search. JZ, QYe, ZW, and LH acquired the data. All authors wrote the manuscript, performed data analysis, revised the manuscript and gave the final approval of the version to be submitted, read and approved the final manuscript.

## Conflict of Interest

The authors declare that the research was conducted in the absence of any commercial or financial relationships that could be construed as a potential conflict of interest.

## Footnotes

1 https://bioconductor.org/packages/impute/

2 http://mirdb.org/

3 https://kmplot.com/analysis/

4 http://www.pancreaticcancerdatabase.org/

## Supplementary Material

TABLE S1 The clinical information of the patients.

TABLE S2 The predicted targets of the 13 identified miRNAs in miRDB.

TABLE S3 The 432 operability signature miRNAs.
